# Relative importance and interactions of factors influencing low-value care provision: a factorial survey experiment among Swedish primary care physicians

**DOI:** 10.1136/bmjqs-2024-018045

**Published:** 2025-02-13

**Authors:** Marta Roczniewska, Hanna Augustsson, Sara Ingvarsson, Emma Hedberg Rundgren, Kamil Szymański, Ulrica von Thiele Schwarz, Per Nilsen, Henna Hasson

**Affiliations:** 1 Department of Learning, Informatics, Management and Ethics, Karolinska Institutet, Stockholm, Sweden; 2 Institute of Psychology, SWPS University, Warszawa, Poland; 3 Center for Epidemiology and Community Medicine, Stockholms Lans Landsting, Stockholm, Sweden; 4 Department of Clinical Neuroscience, Karolinska Institutet, Stockholm, Sweden; 5 School of Health, Care and Social Welfare, Mälardalens universitet, Västerås, Sweden; 6 Department of Medical and Health Sciences, Linköping University, Linköping, Sweden

**Keywords:** Decision making, Primary care, Implementation science

## Abstract

**Background:**

Low-value care (LVC) describes practices that persist in healthcare, despite being ineffective, inefficient or causing harm. Several determinants for the provision of LVC have been identified, but understanding how these factors influence professionals’ decisions, individually and jointly, is a necessary next step to guide deimplementation.

**Methods:**

A factorial survey experiment was employed using vignettes that presented hypothetical medical scenarios among 593 Swedish primary care physicians. Each vignette varied systematically by factors such as patient age, patient request for the LVC, physician’s perception of this practice, practice cost to the primary care centre and time taken to deliver it. For each scenario, we measured the reported likelihood of providing the LVC. We collected information on the physician’s worry about missing a serious illness.

**Results:**

Patient requests and physicians’ positive perceptions of the practice were the factors that increased the reported likelihood of providing LVC the most (by 14 and 13 percentage points (pp), respectively). When the LVC was low in cost or not time-consuming, patient requests further boosted the likelihood of provision by 29 and 18 pp. In contrast, credible evidence against the LVC reduced the role of patient requests by 11 pp. Physicians’ fear of missing a serious illness was linked with higher reported probability of providing LVC, and the credibility of the evidence against the LVC reduced the role of this concern.

**Conclusions:**

The findings highlight that patient requests enhance the role of many determinants, while the credibility of evidence diminishes the impact of others. Overall, these findings point to the relevance of increased clinician knowledge about LVC, tools for patient communication and the use of decision support tools to reduce the uncertainty in decision-making.

**WHAT IS ALREADY KNOWN ON THIS TOPIC**

- Several factors may influence a healthcare professional’s decision about using or abandoning low-value care (LVC) practices, but it is unclear which are more important and how these factors interact to collectively impact decisions.

**WHAT THIS STUDY ADDS**

- Our experimental vignette study showed that patient request and physicians’ positive perception of the practice are the strongest predictors of providing LVC, and the patient requesting the practice amplifies the role of other determinants.
- When there is credible evidence against the LVC practice or this practice is costly, healthcare professionals are less likely to provide it even when patient request is present.

**HOW THIS STUDY MIGHT AFFECT RESEARCH, PRACTICE OR POLICY**

- These findings suggest that increased knowledge about LVC, tools for patient communication and employing decision support tools could help reduce the uncertainty in decision-making among the professionals.

## Background

 Low-value care (LVC) refers to ineffective, inefficient or unwanted care that is unlikely to benefit patients due to potential harms, costs, available alternatives or patient preferences.^1^ Common examples of LVC are unnecessary imaging for low back pain, preoperative laboratory tests^2^ or prescription of antibiotics for respiratory tract infections.^3^ LVC is common, with an estimated prevalence found within 10–30% of all healthcare services,^4 5^ and thus consumes valuable time and resources from strained healthcare system.^6^ LVC also impacts beyond the healthcare system by contributing to carbon emission, waste and pollution.^7^ Both national and international initiatives have been developed to reduce the LVC provision, including Choosing Wisely. The campaign started in the USA and has spread to over 20 countries, aiming to promote the dialogue between healthcare providers and patients about what care is necessary and beneficial. Choosing Wisely also focuses on developing recommendations for such practices. However, even when a practice has been identified as LVC, reducing it has proven to be difficult; thus, solely relying on recommendations is not sufficient.^8^

There are several determinants impacting the use of LVC.^9^,^11^ The amount and quality of the scientific evidence are fundamental to healthcare decisions. Clear evidence against a practice, combined with the availability of an alternative, leads to reduced LVC provision.^12 13^ Beyond the evidence, factors related to patients, economic issues, existing regulations and professionals’ personal knowledge and attitudes all play a role in the decision-making process regarding LVC.^9^,^11^ Among these factors, patient-related aspects,^9^ their expectations and requests are commonly reported to impact the provision of LVC.^14^ Furthermore, patient age has been linked to the provision of LVC. While older age is often associated with increased LVC, the results are not coherent.^15^,^17^ Provision of LVC has also been explained by professionals’ individual characteristics and preferences. Particularly, their experiences of the practice, their conviction for its effects and fear of malpractice have been identified as factors contributing to provision of LVC.^18^ Furthermore, the conditions of the healthcare context also impact the decisions of what practices are provided (eg, steering mechanisms, including financial aspects). There is a risk that professionals are encouraged to choose practices based on reimbursement levels rather than the scientific evidence^19^ and that, among LVC practices, low-cost services are more likely to be delivered than high-cost services.^20^ Time pressure and lack of time for shared decision-making have been reported to increase the likelihood of providing LVC.^21^

Understanding the potential determinants for provision of LVC is important, but it is not sufficient to fully grasp how professionals make decisions regarding LVC in their practice. It is essential to understand how they weigh these different factors and how these factors collectively impact decisions that lead either to the abandonment or continuation of LVC. One of the main knowledge gaps in deimplementation is understanding how professionals navigate the tension between scientific knowledge and patient requests for specific practices. The aim of this study is to experimentally test the influence of various determinants on primary care physicians’ decision-making regarding the provision of LVC. Three research questions (RQs) were formulated:

RQ1. What is the relative importance of determinants associated with the evidence, patients, physicians and healthcare context for physicians’ decisions to provide LVC?

RQ2. How do patients’ requests for LVC and physicians’ perceptions of the credibility of evidence against the requested practice interact with other determinants to affect physicians’ decisions to provide LVC?

RQ3. How does a physician’s concern about missing a serious illness influence their decision to provide LVC?

## Method

### Design

A cross-sectional factorial survey experiment was conducted to investigate the decision-making processes of primary care physicians (for study protocol see ref 22). The participants were exposed to multiple vignettes, that is, hypothetical stories describing a medical scenario concerning a hypothetical LVC, and were asked to make decisions about each one. Online supplemental material 1 presents the extended Consolidated Standards of Reporting Trials checklist for reporting of factorial randomised trials.^23^

### Setting of the study

This research was conducted among primary care physicians in Sweden. Swedish primary healthcare is part of the decentralised tax-funded healthcare system. The responsibility for organising and delivering primary healthcare services lies with the 21 regions.^24^ Primary healthcare is delivered by both public and private service providers who have a care agreement with the region. The providers are funded and governed according to the same rules but can differ in how they are organised and managed.^25^ Primary care comprises around 17% of all healthcare in Sweden and encompasses around 1200 healthcare centres (of which 60% are owned by the regions). Provider fees are set by each region and vary between 60% and 95% in fixed capitation payment, 5–38% in fee-for-service payment and 0–3% in performance-related payment. In Sweden, primary care physicians are specialists in general medicine/family medicine.^26^ They are typically salaried employees, not independent contractors, with the provider fee operating at the primary care centre (PCC) level, not affecting individual physicians.

### Study materials

#### Vignettes

**Figure 1:**
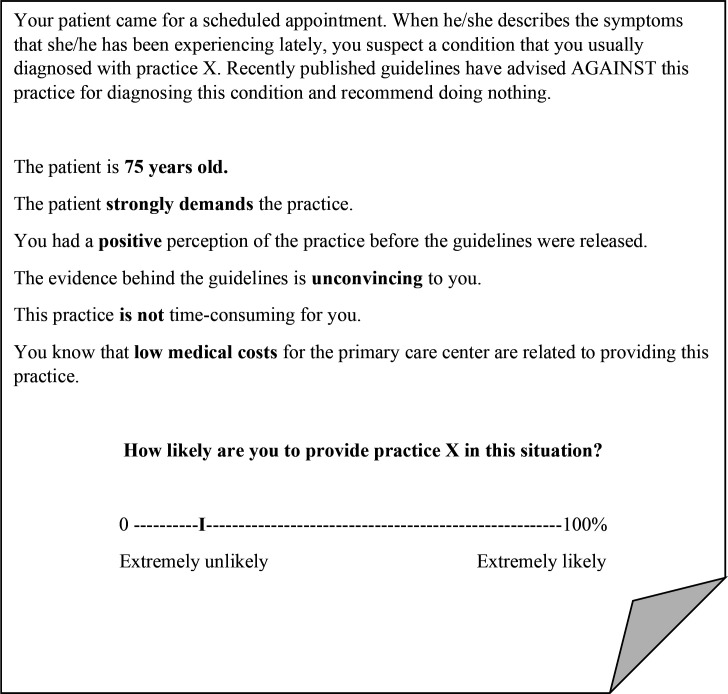
The ‘І’ symbol represents the point that participants could move along the slider to report their response. The vignette was originally presented to participants in Swedish; this is the English translation.

Each vignette simulated a scheduled patient appointment in which the patient described their symptoms, and the physician would typically diagnose them using an unspecified practice. The vignette described how recently published guidelines have advised against this practice in this condition and recommended not to do anything. This part was fixed across the conditions. Then, a randomised combination of factors known to influence the decision to use LVC was fed into each vignette to create hypothetical scenarios. The initial list of factors was chosen based on previously identified determinants of LVC provision.^9 10 27^ Determinants were discussed among the authors based on feasibility for a vignette scenario, that is, (a) forming distinct levels (eg, high vs low cost of the practice), (b) not being a predominating factor (eg, the practice causing harm to patients), (c) appropriate to vary from a respondent perspective (eg, patient age rather than physician’s own characteristics such as tenure) and (d) ecologically valid (ie, applicable in Swedish primary care). Development of the vignettes and pilot-testing procedure are described in detail in online supplemental material 2. In the final version, the following factors were included: patient age, patient request, the physician’s perception of the practice, credibility of the evidence against the practice, time consumption for the physician associated with the practice and costs for the PCC (see table 1). Figure 1 demonstrates an example of a vignette in English, with varied dimensions bolded. After being provided with the vignette text, the physicians were asked to rate how likely they were to provide the practice advised against on a scale from 0% (*extremely unlikely*) to 100% (*extremely likely*) using a slider to mark their answer.

**Table 1:** Vignette dimensions and their levels

Dimensions	Levels		
	1	2	3
Patient age	25	50	75
Patient request	Absent (neutral towards the practice)	Present (strongly requests the practice)	–
Physician’s perception of the practice	Negative	Positive	–
Credibility of the evidence against the practice	Unconvincing	Convincing	–
Time consumption associated with the practice for the physician	Not time-consuming	Time-consuming	–
Costs for the primary care centre	Low	High	–

The vignette universe was generated by crossing the possible combinations (Cartesian product) of the above vignette dimensions and their levels to warrant orthogonality across the factors (3×2×2×2×2×2), resulting in 96 vignettes. Given the number of dimensions, their levels and the two-way interactions we wanted to test, we took a subsample of 24 vignettes which provided a D-efficiency of 98/100. We applied the SAS macro ‘%MktBlock’ to ensure a randomised distribution of the 24 vignettes over four versions of the survey (decks). Each deck comprised six vignettes. Online supplemental material 3 details the applied fractional factorial design, method for vignette selection and their blocking into decks.

### Experimental procedure

Participants were primary care physicians recruited in 2023 through various channels: representatives from primary care organisations in Sweden, a newsletter from the national organisation for primary care physicians and Facebook groups for physicians. This recruitment method was supported by a social media campaign (paid advertising) aiming to target primary care physicians. The invitation included a link to a web page that explained the purpose of our research and contained a link to the survey. Participants could download a file containing information about their rights, data management, etc. After reading this information, individuals provided informed consent to participate. Participants were randomly assigned to one of the four decks. Participants were informed that they would be asked to evaluate a series of vignettes that described a hypothetical medical scenario, and that they would need to decide in each case. Every participant read six scenarios. The vignettes were presented one at a time in a randomised order. After rating all vignettes, physicians answered background questions about themselves and their work, and reported the extent to which they worried that they would miss a serious illness when diagnosing and treating their patients.

### Analytical strategy

We performed multilevel modelling (MLM) to test how the outlined factors affect physicians’ decision-making. The data structure was hierarchical: the vignette factors were entered as level 1 variables, while variables related to the respondent were entered as level 2 variables.

First, a random intercept-only model with no predictors (model 0) was created to calculate an intraclass correlation coefficient (ICC) and to benchmark model fit. This model contained a random intercept related to the participant and the vignette deck. To answer RQ1, in model 1, variables related to vignette dimensions were entered to test the main effects of each factor. To answer RQ2, in model 2, we added the interactions of patient request with all other factors, and credibility of evidence with all other factors. For significant interactions, we performed pairwise comparisons with Bonferroni corrections (see online supplemental material 4). To answer RQ3, in model 3, we added the variable measuring the concern about missing a serious illness in the diagnosis. This variable was z-scored. We also tested whether being worried moderated the effect of patient request and credibility of evidence on the decisions. To control the physicians’ characteristics, we added their gender and work experience. Across all models, we kept the random intercept of the respondent and the deck. Due to the complexity of modelling this number of interactions, we did not enter any random slopes. When comparing the conditions, throughout the results, we refer to absolute change in reported probabilities of providing LVC, that is, percentage points (pp). The analyses were performed in JAMOVI.^28^

## Results

### Respondents

A total of 593 individuals provided consent to participate (393 women, 173 men, 2 persons who identified as non-binary and 25 persons who did not disclose their gender). The majority were individuals aged 30–39 (37.2%) and 40–49 (35.8%) years, followed by those in the range of 50–59 (14.9%) and 18–29 (9.7%), while 2.4% of the sample were aged 60 and above. Participants represented all 21 healthcare regions in Sweden (see online supplemental material 5). Most were employed by a public provider (61.8%), others worked for private providers (35.6%) and some shared their work time between public and private (2.6%). Eight per cent had worked for less than 1 year, 15.3% between 1 and 2 years, 27.4% between 3 and 5 years, 21% between 6 and 10 years and 28.3% for more than 10 years. The mean self-reported concern about missing a serious illness when diagnosing and treating their patients was 2.20 (SD=1.10) on a scale from 1 (*definitely not*) to 5 (*definitely yes*).

### Main findings

Table 2 presents the results of the multilevel analysis, with the reported probability of providing the LVC practice as an outcome (0–100%). Model 0 is a two-level intercept-only model with random intercept related to the physician (ICC=0.23) and the vignette deck (ICC=0.02), justifying the need for MLM to control for person effects.

**Table 2:** Multilevel analysis predicting the self-rated likelihood of providing the LVC from vignette (level 1) and participant (level 2) factors

Predictors	Model 0		Model 1		Model 2		Model 3	
	Est	95% CI	Est	95% CI	Est	95% CI	Est	95% CI
Intercept	27.30***	23.90 to 30.60	24.67**	16.76 to 32.58	25.11*	10.45 to 39.77	24.59*	10.66 to 38.52
**Level 1**								
Patient age (AGE)								
50–25 (contrast)			3.96***	1.52 to 4.60	4.12**	1.54 to 6.70	3.85**	1.23 to 6.46
75–25 (contrast)			6.00***	4.47 to 7.53	6.13***	3.73 to 8.54	6.01***	3.56 to 8.46
Patient request (REQUEST)†			13.86***	12.49 to 15.24	15.56***	13.59 to 17.52	15.38***	13.37 to 17.39
Physician’s prior perception of the practice (PERCEPTION)‡			13.18***	11.97 to 14.40	14.20***	12.98 to 15.43	14.32***	13.08 to 15.57
Credibility of the evidence (EVIDENCE)§			−7.08***	−8.32 to −5.85	−7.30***	−9.15 to −5.44	−7.46***	−9.35 to −5.57
Time consumption associated with the practice for the physician (TIME)¶			−6.18***	−7.42 to −4.94	−5.62***	−7.84 to −3.41	−5.47***	−7.72 to −3.21
Costs for the primary care centre (COST)††			−11.24***	−12.68 to −9.80	−4.65*	−8.53 to −0.77	−5.35**	−9.29 to −1.41
Interactions								
REQUEST×AGE 50–25 (contrast)					−8.66	−19.23 to 1.91	−6.80	−17.52 to 3.92
REQUEST×AGE 75–25 (contrast)					−19.00***	−28.95 to −9.06	−16.84**	−26.94 to −6.74
REQUEST×PERCEPTION					−5.65*	−10.35 to −0.96	−6.35**	−11.11 to −1.59
REQUEST×EVIDENCE					−7.53***	−11.22 to −3.85	−7.10***	−10.84 to −3.37
REQUEST×TIME					−26.15***	−35.17 to −17.13	−24.60***	−33.75 to −15.45
REQUEST×COST					−4.31**	−7.32 to −1.31	−4.75**	−7.82 to −1.68
EVIDENCE×AGE 50–25 (contrast)					−21.36***	−28.44 to −14.28	−19.61***	−26.80 to −12.43
EVIDENCE×AGE 75–25 (contrast)					3.28	−2.40 to 8.95	3.14	−2.64 to 8.91
EVIDENCE×PERCEPTION					−4.25	−9.62 to 1.12	−5.06	−10.51 to 0.40
EVIDENCE×TIME					−31.67***	−49.10 to −14.25	−28.77**	−46.48 to −11.05
EVIDENCE×COST					−1.40	−5.58 to 2.78	−1.43	−5.68 to 2.83
**Level 2**								
Physician gender‡‡							−1.11	−3.86 to 1.65
Physician tenure								
Less than 1 year—more than 10 years (contrast)							−2.45	−7.45 to 2.56
1–2 years—more than 10 years (contrast)							−2.21	−6.19 to 1.78
3–5 years—more than 10 years (contrast)							1.02	−2.31 to 4.35
6–10 years—more than 10 years (contrast)							1.36	−2.17 to 4.89
Physician’s worry to miss a serious illness (WORRY)							3.59***	2.30 to 4.88
**Cross-level interactions**								
REQUEST×WORRY							0.81	−0.44 to 2.07
EVIDENCE×WORRY							−1.47*	−2.67 to −0.27
**Random effects**								
σ^2^	511.20		337.50		316.00		312.00	
τ_00 person_	152.52		181.10		185.00		167.00	
τ_00 deck_	9.88		63.50		222.00		197.00	
ICC_person_	0.23		0.35		0.37		0.35	
N_person_	593		593		593		566	
Observations	3552		3552		3552		3391	
Marginal R^2^/conditional R^2^	0.000/0.241		0.220/0.547		0.282/0.686		0.300/0.676	
Log likelihood	−16 422.43		−15 803.24		−15 683.14		−14 916.75	
AIC	32 855.60		31 640.17		31 456.71		29 956.47	
BIC	32 877.43		31 696.41		31 546.14		30 062.22	

CI Method: Wald. *** *p* < 0.001, ** *p* < 0.01, * *p* < 0.05

† 0 – absent, 1 – present

‡ 0 – negative, 1 – positive

§ 0 – unconvincing, 1 – convincing

¶ 0 – not time-consuming, 1 – time-consuming

†† 0 – low, 1 – high

‡‡ 0 – man, 1 – woman

Est, estimate; ICC, intraclass correlation coefficient; LVC, low-value care; σ2, residual variance; τ_00_, random intercept variance.

#### RQ1. What is the relative importance of determinants associated with the evidence, patients, physicians and healthcare context for physicians’ decisions to provide LVC?

The results demonstrated that patient request for the practice and the physician’s perception of the practice were predictors of the largest magnitude for the physician’s decision to provide the LVC practice. Specifically, we found that a requesting patient increased the reported probability of providing the LVC by 14 pp compared with when no request was made. A physician’s positive perception of the practice was linked with a 13 pp higher reported probability of providing the LVC compared with a negative perception. The findings showed that a low (vs high) cost of the practice to PCC increased the reported probability of providing the LVC practice by 11 pp. A practice that was not time-consuming increased the reported probability of providing it by 6 pp compared with one that was time-consuming. Conversely, when scientific evidence behind refraining from the practice was credible, it decreased the reported probability of providing the LVC by 7 pp. When it comes to patient age, a 25-year-old patient was less likely to be provided with LVC than a 50-year-old or a 75-year-old patient.

#### RQ2. How do patients’ requests for LVC and physicians’ perceptions of the credibility of evidence against the requested practice interact with other determinants to affect physicians’ decisions to provide LVC?

**Figure 2:**
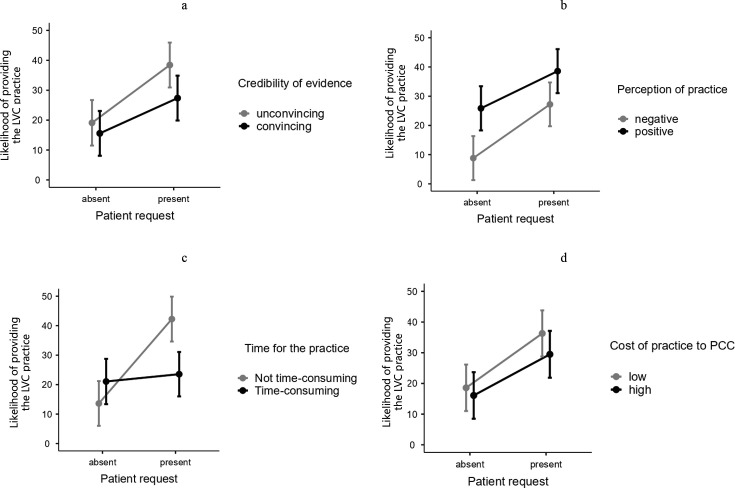
Y-axis refers to self-reported percentage likelihood of providing the low-value care (LVC) practice on a scale from 0% (extremely unlikely) to 100% (extremely likely). Error bars represent SEs. PCC, primary care centre.

The patient request factor significantly interacted with all other factors (see figure 2a–d).

An interaction between patient request and credibility of evidence (figure 2a) demonstrated that while the requesting patient increased the reported probability of providing LVC compared with a neutral one, it did it much less when evidence behind refraining from the practice was convincing (12 pp) than when it was not convincing (19 pp). The credibility of the evidence lowered the reported probability of providing LVC, especially in cases of a demanding patient (11 pp).

An interaction between patient request and the physician’s perception of the practice (figure 2b) demonstrated that the reported probability of providing LVC was lowest when the physician had a negative attitude to the practice and there was no request from the patient, while it was highest when patient request was accompanied by the physician’s positive attitude towards the practice.

The interaction between patient request and time consumption associated with the practice (figure 2c) demonstrated that when the practice was time-consuming, the request from the patient did not affect the physician’s decision. However, when the practice was not time-consuming, the requesting patient increased the reported probability of providing the practice by over 29 pp, compared with a lack of such a request.

An interaction between patient request and the cost of the practice for PCC (figure 2d) showed that in cases of no request from the patient, the costs did not affect the physician’s decision. A request for the practice from the patient increased the reported probability of providing LVC in contrast to a lack of such request from the patient, but it did so more for practices that were low in cost (18 pp) compared with those high in cost (13 pp).

The interaction between patient request and age showed that in cases of a patient requesting the practice, age did not affect the physician’s decision. However, when the patient was neutral towards the practice, a 75-year-old patient was more likely be provided with LVC compared with a 50-year-old or 25-year-old patient.

**Figure 3:**
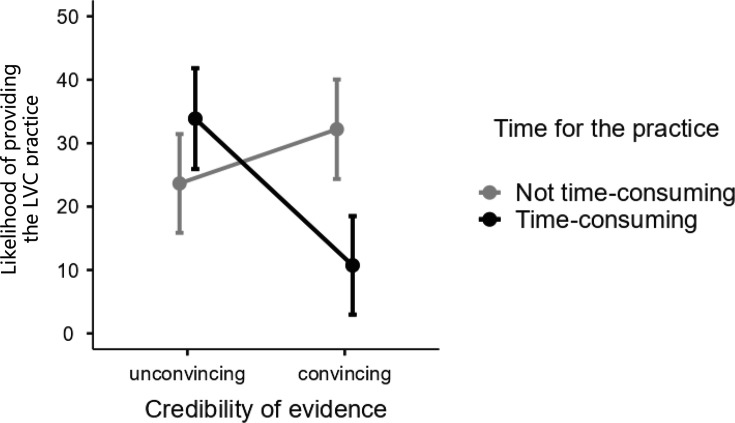
Y-axis refers to self-reported percentage likelihood of providing the low-value care (LVC) practice on a scale from 0% (extremely unlikely) to 100% (extremely likely). Error bars represent SEs.

We also observed an interaction between the credibility of the evidence and the cost of the practice for PCC (see figure 3). The findings showed that convincing evidence combined with a time-consuming practice significantly lowered the reported probability of providing LVC compared with other conditions.

#### RQ3. How does a physician’s concern about missing a serious illness influence their decision to provide LVC?

Self-reported concern about missing a serious diagnosis was significantly linked with the physician’s decision to provide LVC: the more concerned the physician reported to be about missing a serious illness, the higher was their reported probability of providing LVC. We also tested whether the physician’s concern moderated the effect of patient request or credibility of evidence on their decision to provide LVC. While there was no interaction with a patient request, we observed one regarding the credibility of evidence: although the reported probability of providing LVC grew as the concern increased, the slope was less steep in cases of convincing evidence compared with unconvincing evidence.

## Discussion

We found that all of the examined factors (ie, patient age and request, credibility of the evidence, the physician’s perception of the practice, time consumption associated with the practice for the physician and cost of the practice to the PCC) individually contributed to the reported probability of using the LVC. Of these, a patient’s request and a physician’s positive perceptions of the practice were the most influential factors when it comes to the magnitude of the effects. In addition, we found that a patient’s request amplified the role of low cost and short time of providing LVC for the physician’s decision. On the other hand, we found that high credibility of the evidence for the practice being LVC, as well as time-consuming practices, had a buffering role against patient request. Finally, we observed that physicians who were concerned about missing a serious illness reported higher likelihood of providing the LVC, although this effect was reduced by convincing evidence. Below, we expand on these contributions.

The literature on LVC has shown that factors related to patients are the most commonly identified determinants of LVC.^9^ Our study adds to this by demonstrating that in this experimental study of a limited number of determinants, patient request for a practice appears to strongly drive physicians’ decision to provide LVC, compared with the other studied factors. Additionally, we contribute to the literature by showing that patient request also amplifies the role of other determinants such as when the LVC comes at a low cost or is not time-consuming. Thus, when LVC is requested by the patient, other determinants further increase the likelihood of its provision. However, more research is needed to understand why, how and under what circumstances patient requests amplify the role of other determinants. Future research could investigate whether the various reasons behind patient requests influence the physicians’ decisions to provide a certain treatment or test differently. Additionally, physicians may adhere to patients’ requests for LVC to make patients satisfied with the care.^27^ However, the relationship between providing LVC and patients’ satisfaction is unclear. While there is research demonstrating that clinician denial of requests predicts lower patient satisfaction when compared with fulfilment of such requests,^29^ others found that providing LVC had no or minimal impact on patient’s experience rating.^30 31^ Nevertheless, if physicians believe that denying patient requests will have a negative impact on patients’ experience ratings, the role of patient requests for LVC may be even more prominent in settings where patient satisfaction scores affect physicians’ compensation or the economics of the healthcare organisation. We also found that the role of patient request for the physician’s decision may be mitigated by convincing evidence against the practice, and the fact that the practice is time consuming. Situations when patients request the practice increase physicians’ likelihood of providing it, but less so when the evidence against it is convincing to the physician. Thus, while it is not sufficient to ensure there is credible evidence to discourage LVC, as this on its own is a less important factor than patient request, convincing evidence can still play a buffering role in situations when patients request the practice. We found that the time factor has a stronger buffering role than the credibility of evidence: when the practice is time consuming for the physicians, it lowers the reported probability to provide it to a similar level that it would be with patients who are neutral to the practice. Finally, we observed that physicians who experience more concern about missing a serious illness are more likely to report higher probability of providing the LVC practice. Yet, when the evidence is convincing, the concern contributes less to the decision compared with unconvincing evidence.

Our research offers valuable insights into primary care physicians’ decision-making concerning LVC, particularly concerning the dynamics between patient request for a practice and the evidence supporting the need to refrain from it. The findings have numerous practical implications. First, the role of patient request that we uncovered suggests a need for strategies to manage patient expectations. These could relate to efforts to inform and educate the public^32^ about the risks of certain practices as patients tend to overestimate the benefits of treatments or tests, and underestimate the harms.^33^ Contrary to common assumptions among clinicians, unnecessary testing does not necessarily reassure worried patients.^34^ Suggested strategies instead involve different types of communication tools,^35 36^ including scripts for the clinicians on how to clearly describe why they are not providing a practice while maintaining an empathetic approach.^37^ Furthermore, clearer guidelines for physicians regarding which practices are considered LVC, in combination with feedback on use, could counterbalance the impact of patient requests.^36^ A strategy that could counteract for the time factor could be so-called accountability tools, where clinicians are asked to provide an argument for using LVC (eg, in the electronic health record).^35^ This provides an opportunity to reflect on the reason, but also takes time, which might lead to not providing it at all. Furthermore, the finding that convincing evidence can reduce the likelihood of physicians providing LVC in the face of patient demands or the physician’s concern highlights the importance of continuous professional training on research and guidelines.^38^ However, merely relying on continuous education is not sufficient as one challenge in the deimplementation of LVC is the rapidly evolving evidence and the risk of information overload.^39^ Therefore, systems such as decision support tools^38^ are important to make physicians feel more confident in their decisions, particularly when facing pressure from patients or when they are concerned about missing a serious illness.

### Strengths and limitations

The strengths of this research include the use of experimental design, where multiple factors were tested to isolate and juxtapose their effects. Clinicians were consulted on the vignettes, which were designed to reflect the real-world scenarios in a controlled way; by presenting the same story to different participants, we ensured a level of standardisation that is hard to achieve in observational studies and makes the responses more appropriate for comparisons across the scenarios. Another strength is the fact that taking an experiment approach allowed us to examine the decisions without putting patients at risk or facing other ethical complexities.

The study has limitations that need to be acknowledged. First, the physicians took their decisions in imaginary scenarios and not actual cases. Thus, we capture their perceptions of what they *would do* rather than actual decisions that they make in their consulting rooms. Yet, factorial survey experiments have been particularly suitable for investigating complex or sensitive situations with multiple factors affecting judgements.^40^ Professionals’ decision-making is challenging to study using conventional methods (interviews, observations), which could be prone to social desirability. Second, because the scenarios in the vignettes should not contain an excessive number of factors, we limited our investigations to the most prominent determinants. This means that some relevant factors^9^,^11^ have not been included. Third, while the physicians who took part in this study were employed in all 21 regions in Sweden, the recruitment method did not ensure a representative sample when it comes to region, gender, age or work experience. In addition, the focus was by design on physicians in primary care to achieve high validity of the scenario and factors; however, it also limits the generalisability to other medical specialties and settings.

## Conclusions

The study contributes to an understanding of factors influencing primary care physicians’ decisions to provide LVC practices despite recommendations to not do anything. Among the six examined factors, patient request and physicians’ positive perceptions of the practice impacted physicians’ decisions the most when analysed in isolation. Patient request amplifies the role of other determinants of LVC provision, such as low cost of the practice or the fact that it is not time-consuming. Conversely, perceived credibility of the evidence behind the recommendations and time consumption associated with performing the practice act as buffers against patient request. Overall, these findings point to the relevance of enhanced knowledge about LVC, tools for patient communication and the use of decision support tools to reduce the uncertainty in decision-making.

## Supplementary material

10.1136/bmjqs-2024-018045 online supplemental material 1

10.1136/bmjqs-2024-018045 online supplemental material 2

10.1136/bmjqs-2024-018045 online supplemental material 3

10.1136/bmjqs-2024-018045 online supplemental material 4

10.1136/bmjqs-2024-018045 online supplemental material 5

## Data Availability

Data are available upon reasonable request.

## References

[1] Verkerk EW, Tanke MAC, Kool RB (2018). Limit, lean or listen? A typology of low-value care that gives direction in de-implementation. Int J Qual Health Care.

[2] Müskens JLJM, Kool RB, van Dulmen SA (2022). Overuse of diagnostic testing in healthcare: a systematic review. BMJ Qual Saf.

[3] Magin P, Tapley A, Morgan S (2018). Reducing early career general practitioners’ antibiotic prescribing for respiratory tract infections: a pragmatic prospective non-randomised controlled trial. Fam Pract.

[4] Brownlee S, Chalkidou K, Doust J (2017). Evidence for overuse of medical services around the world. Lancet.

[5] Grimshaw JM, Patey AM, Kirkham KR (2020). De-implementing wisely: developing the evidence base to reduce low-value care. BMJ Qual Saf.

[6] Korenstein D, Chimonas S, Barrow B (2018). Development of a Conceptual Map of Negative Consequences for Patients of Overuse of Medical Tests and Treatments. JAMA Intern Med.

[7] Braithwaite J, Pichumani A, Crowley P (2023). Tackling climate change: the pivotal role of clinicians. BMJ.

[8] Rosenberg A, Agiro A, Gottlieb M (2015). Early Trends Among Seven Recommendations From the Choosing Wisely Campaign. JAMA Intern Med.

[9] Augustsson H, Ingvarsson S, Nilsen P (2021). Determinants for the use and de-implementation of low-value care in health care: a scoping review. *Implement Sci Commun*.

[10] van Dulmen SA, Naaktgeboren CA, Heus P (2020). Barriers and facilitators to reduce low-value care: a qualitative evidence synthesis. BMJ Open.

[11] Leigh JP, Sypes EE, Straus SE (2022). Determinants of the de-implementation of low-value care: a multi-method study. BMC Health Serv Res.

[12] Specchia ML, La Torre G, Calabrò GE (2018). Disinvestment in cancer care: a survey investigating European countries’ opinions and views. Eur J Public Health.

[13] Voorn VMA, Marang-van de Mheen PJ, Wentink MM (2014). Perceived barriers among physicians for stopping non-cost-effective blood-saving measures in total hip and total knee arthroplasties. Transfusion.

[14] Raudasoja A, Tikkinen KAO, Bellini B (2024). Perspectives on low-value care and barriers to de-implementation among primary care physicians: a multinational survey. *BMC Prim Care*.

[15] Faustino CG, Martins M de A, Jacob Filho W (2011). Potentially inappropriate medication prescribed to elderly outpatients at a general medicine unit. Einstein (Sao Paulo).

[16] Bhatia RS, Bouck Z, Ivers NM (2017). Electrocardiograms in Low-Risk Patients Undergoing an Annual Health Examination. JAMA Intern Med.

[17] Dallas A, Magin P, Morgan S (2015). Antibiotic prescribing for respiratory infections: a cross-sectional analysis of the ReCEnT study exploring the habits of early-career doctors in primary care. Fam Pract.

[18] Bishop TF, Cea M, Miranda Y (2017). Academic physicians’ views on low-value services and the choosing wisely campaign: A qualitative study. Healthcare (Basel).

[19] Alber K, Kuehlein T, Schedlbauer A (2017). Medical overuse and quaternary prevention in primary care - A qualitative study with general practitioners. BMC Fam Pract.

[20] Mafi JN, Russell K, Bortz BA (2017). Low-Cost, High-Volume Health Services Contribute The Most To Unnecessary Health Spending. Health Aff (Millwood).

[21] Kool RB, Verkerk EW, Winnemuller LJ (2020). Identifying and de-implementing low-value care in primary care: the GP’s perspective-a cross-sectional survey. BMJ Open.

[22] Roczniewska M, von Thiele Schwarz U, Augustsson H (2021). How do healthcare professionals make decisions concerning low-value care practices? Study protocol of a factorial survey experiment on de-implementation. Implement Sci Commun.

[23] Kahan BC, Hall SS, Beller EM (2023). Reporting of Factorial Randomized Trials: Extension of the CONSORT 2010 Statement. JAMA.

[24] Glenngård AH (2020). International health care system profiles—sweden. https://www.commonwealthfund.org/international-health-policy-center/countries/sweden.

[25] Glenngård AH (2023). Exploring differences between public and private providers in primary care: findings from a large Swedish region. HEPL.

[26] Bexelius TS, Olsson C, Järnbert-Pettersson H (2016). Association between personality traits and future choice of specialisation among Swedish doctors: a cross-sectional study. Postgrad Med J.

[27] Ingvarsson S, Augustsson H, Hasson H (2020). Why do they do it? A grounded theory study of the use of low-value care among primary health care physicians. Implement Sci.

[28] (2023). The jamovi project. jamovi. https://www.jamovi.org.

[29] Jerant A, Fenton JJ, Kravitz RL (2018). Association of Clinician Denial of Patient Requests With Patient Satisfaction. JAMA Intern Med.

[30] Sanghavi P, McWilliams JM, Schwartz AL (2021). Association of Low-Value Care Exposure With Health Care Experience Ratings Among Patient Panels. JAMA Intern Med.

[31] Rockwell MS, Michaels KC, Epling JW (2022). Does de-implementation of low-value care impact the patient-clinician relationship? A mixed methods study. BMC Health Serv Res.

[32] Sypes EE, de Grood C, Clement FM (2020). Understanding the public’s role in reducing low-value care: a scoping review. Implement Sci.

[33] Hoffmann TC, Del Mar C (2015). Patients’ expectations of the benefits and harms of treatments, screening, and tests: a systematic review. JAMA Intern Med.

[34] Rolfe A, Burton C (2013). Reassurance after diagnostic testing with a low pretest probability of serious disease: systematic review and meta-analysis. JAMA Intern Med.

[35] Ingvarsson S, Hasson H, von Thiele Schwarz U (2022). Strategies for de-implementation of low-value care-a scoping review. Implement Sci.

[36] Ingvarsson S, Sandaker I, Nilsen P (2023). Strategies to reduce low-value care - An applied behavior analysis using a single-case design. Front Health Serv.

[37] Takada T, Heus P, van Doorn S (2020). Strategies to reduce the use of low-value medical tests in primary care: a systematic review. Br J Gen Pract.

[38] Heus P, van Dulmen SA, Weenink J-W (2023). What are Effective Strategies to Reduce Low-Value Care? An Analysis of 121 Randomized Deimplementation Studies. J Healthc Qual.

[39] Shepperd S, Adams R, Hill A (2013). Challenges to using evidence from systematic reviews to stop ineffective practice: an interview study. J Health Serv Res Policy.

[40] Auspurg K, Hinz T (2015). Factorial Survey Experiments.

