# Supplementary material for: Relative importance and interactions of factors influencing low-value care provision: a factorial survey experiment among Swedish primary care physicians
Source: BMJ Qual Saf. 2025 Feb 13;34(9):e018045. doi: 10.1136/bmjqs-2024-018045 (PMC12418588; doi:10.1136/bmjqs-2024-018045)
Supplement: online supplemental material 1 [file bmjqs-34-9-s001.pdf]

Supplementary material 1

CONSORT Checklist of Information to Include When Reporting Factorial Randomized Trials

**Section**	**Item No.**	**CONSORT 2010 Statement Checklist Item**	**Extension for Factorial Trials**	**Page number**
Title and Abstract				
Title	1a	Identification as a randomized trial in the title	Identification as a factorial randomized trial in the title	1
Abstract	1b	Structured summary of trial design, methods, results, and conclusions (for specific guidance see CONSORT for abstracts)	See separate factorial checklist for abstracts	2
Introduction				
Background	2a	Scientific background and explanation of rationale	Scientific background and rationale for using a factorial design, including whether an interaction is hypothesized	4—5
Objectives	2b	Specific objectives or hypotheses	Specific objectives or hypotheses and a statement of which treatment groups form the main comparisons	5—6
Methods				
Trial design	3a	Description of trial design (such as parallel, factorial) including allocation ratio	Description of the type of factorial trial (such as full or partial, number of factors, levels within each factor) and allocation ratio	8
Change from protocol	3b	Important changes to methods after trial commencement (such as eligibility criteria), with reasons		NA
Participants	4a	Eligibility criteria for participants	Eligibility criteria for each factor,	8

			noting any differences, if applicable	
Setting and location	4b	Settings and locations where the data were collected		6
Interventions	5	The interventions for each group with sufficient details to allow replication, including how and when they were actually administered		NA
Outcomes	6a	Completely defined pre-specified primary and secondary outcome measures, including how and when they were assessed		7
Changes to outcomes	6b	Any changes to trial outcomes after the trial commenced, with reasons		NA
Sample size	7a	How sample size was determined	How sample size was determined for each main comparison, including whether an interaction was assumed in the calculation	10
Interim analyses and stopping guidelines	7b	When applicable, explanation of any interim analyses and stopping guidelines	When applicable, explanation of any interim analyses and stopping guidelines, noting any differences across main comparisons and reasons for differences	NA
Randomization				
Sequence generation	8a	Method used to generate the random allocation sequence		8; Supplementary material 3
Sequence generation	8b	Type of randomization; details of any	Type of randomization, details of any	8; Supplementary material 3

		restriction (such as blocking and block size)	restriction (such as blocking and block size), and, if applicable, whether participants were randomized to factors at different time points	
Allocation concealment mechanism	9	Mechanism used to implement the random allocation sequence (such as sequentially numbered containers), describing any steps taken to conceal the sequence until interventions were assigned		NA
Implementation	10	Who generated the random allocation sequence, who enrolled participants, and who assigned participants to interventions		NA
Blinding	11a	If done, who was blinded after assignment to interventions (for example, participants, care providers, those assessing outcomes)		NA
Similarity of interventions	11b	If relevant, description of the similarity of interventions		NA
Statistical methods	12a	Statistical methods used to compare groups for primary and secondary outcomes	Statistical methods used for each main comparison for primary and secondary outcomes, including:<br>- Whether the target treatment effect for each main	9

			comparison pertains to the effect in the presence or absence of other factors<br>- Approach to analysis, such as factorial or multiarm<br>- How the approach was chosen, such as prespecified or based on estimated interaction<br>- If factorial approach was used, whether factors were adjusted for each other<br>- If applicable, how nonconcurrent recruitment to factors was handled<br>- Method(s) used to evaluate statistical interaction(s)	
Additional analyses	12b	Methods for additional analyses, such as subgroup analyses and adjusted analyses		NA
Results				
Participant flow (a diagram is strongly recommended)	13a	For each group, the numbers of participants who were randomly assigned, received intended treatment, and were analyzed for the primary outcome	For each main comparison, the number of participants who were randomly assigned, received intended treatment, and were analyzed for the primary outcome	NA
Losses and exclusions	13b	For each group, losses and exclusions after randomization, together with reasons	For each main comparison, losses and exclusions after randomization, together with reasons	NA
Recruitment	14a	Dates defining the periods of	Dates defining the periods of recruitment and	8

		recruitment and follow-up	follow-up for each factor, noting any differences, with reasons	
Trial end	14b	Why the trial ended or was stopped		NA
Baseline data	15	A table showing baseline demographic and clinical characteristics for each group	A table showing baseline demographic and clinical characteristics for each main comparison	NA
Numbers analyzed	16	For each group, the number of participants (denominator) included in each analysis and whether the analysis was by original assigned groups	For each main comparison, the number of participants (denominator) included in each analysis and whether the analysis was by original assigned groups	NA
Outcomes and estimation	17a	For each primary and secondary outcome, results for each group and the estimated effect size and its precision (such as 95% CI)	For each primary and secondary outcome, results for each main comparison, the estimated effect size, and its precision (such as 95% CI)<br>For each primary outcome, the estimated interaction effect and its precision<br>If done, the estimated interaction effects and precision for secondary outcomes	10—13<br>Table 2<br>Supplementary material 5
Binary outcomes	17b	For binary outcomes, presentation of both absolute and relative effect sizes is recommended		NA

Ancillary analyses	18a	Results of any other analyses performed, including subgroup analyses and adjusted analyses, distinguishing prespecified from exploratory		NA
Additional data summaries	18b	New item	Participant flow, losses and exclusions, baseline data, and outcome data (including primary and secondary outcomes, harms, and adherence) presented by treatment groups	NA
Harms	19	All important harms or unintended effects in each group (for specific guidance see CONSORT for harms)	All important harms or unintended effects for each main comparison	NA
Discussion				
Limitations	20	Trial limitations, addressing sources of potential bias, imprecision, and, if relevant, multiplicity of analyses		16—17
Generalizability	21	Generalizability (external validity, applicability) of the trial findings		16—17
Interpretation	22	Interpretation consistent with results, balancing benefits and harms, and considering other relevant evidence		13—16
Other Information				
Registration	23	Registration number and name of trial registry		NA

Protocol	24	Where the full trial protocol can be accessed, if available		6
Funding	25	Sources of funding and other support (such as supply of drugs), role of funders		Blinded for peer review
