# Supplementary material for: Relative importance and interactions of factors influencing low-value care provision: a factorial survey experiment among Swedish primary care physicians
Source: BMJ Qual Saf. 2025 Feb 13;34(9):e018045. doi: 10.1136/bmjqs-2024-018045 (PMC12418588; doi:10.1136/bmjqs-2024-018045)
Supplement: online supplemental material 2 [file bmjqs-34-9-s002.pdf]

Supplementary material 2 – Development and pilot-testing of the vignettes

The initial list of factors was chosen based on previously identified determinants of LVC provision [1–3]. All determinants were discussed among authors to judge which were feasible for a vignette scenario, i.e., would be appropriate to manipulate by making it easier to the physician to imagine rather than change their own characteristics (e.g., patient age to be manipulated rather than physician's work experience), could form distinct levels (e.g., high vs low cost of the practice), would not form a predominating factor (such as information about the practice causing harm to patients), and should be ecologically valid (i.e., would be applicable in Swedish primary care).

From the list of determinants concerning patient characteristics (age, gender, and ethnicity [2]), only age was included as it was assumed that that participants would not openly change their answers based on gender or ethnicity bias due to social desirability. Furthermore, based on research demonstrating the important role of expressed patient expectations [1–3], we incorporated patient request as another determinant.

Based on the literature [1], we initially included a factor that related to the trustworthiness of the source of the guidelines. However, the pilot interviews revealed that this factor had low ecological validity as respondents could not imagine that published standards could not be trustworthy. Instead, we introduced a factor that described how convincing the evidence behind the guidelines was.

Next, we considered two determinants for professionals' perceptions of the practice: having a good experience of the practice, and knowledge about the harmful effects of the practice [2]. Having good experience of the practice was added to the vignette because it was assumed that explicit information that the practice was harmful would cancel out all the other factors.

Based on literature reviews [2,3] demonstrating that LVC practices that are relatively cheaper are more likely to be provided than more expensive ones, we included cost of the practice to the healthcare center as another determinant in the vignette. Finally, past research has shown that time pressure is associated with higher use of LVC [2]. However, we deemed that it might be difficult for the respondents to imagine actual time pressure to the point that it affected their decisions. We used a proxy for this factor, where we described the LVC practice in question as time-consuming or not.

The vignette was iteratively pilot-tested with eight primary care physicians, using cognitive interviewing techniques [4]. The interview guide included think aloud and verbal probing techniques, and complementary questions about the experience of responding to the vignette. The physicians were first asked to read the vignettes out loud and to think aloud while providing their answers. They were then asked to describe what each factor meant to them and how they perceived these factors affected the likelihood of providing the LVC. Lastly, they were asked about their experiences of answering the vignette questions and about the phrasing of the factors.

Three rounds of pilot interviews were conducted, with two to three physicians participating in each round. Three of the participants were specialists in general practice, two were interns currently or recently working in general practice, and two were residents from other specialties. At least one physician currently working in primary care was included in each round of pilot interviews.

After each round of interviews, the vignette content was revised based on the participants' interpretation of the factors to ensure that participants interpret each of the factors in the vignette story as intended (e.g. "This practice is not time-consuming for you" interpreted as practice that consumes physicians time to perform rather than the physician experiencing lack of time to perform it). When the vignette structure had been revised, it was

tested again and iteratively revised until the participants interpreted all factors as intended by the research team.

Additionally, we checked the ranges of their responses, depending on the combination of factors. As expected, a vignette with a certain combination of factors (e.g., high requesting patient, low time and low medical consumption of the practice, previous positive attitude of the physician towards this practice, and unconvincing evidence) resulted in higher ranges of responses (15-50%) than the opposite combinations (0-20%). This showed that there were not only individual differences with regards to response patterns, but also that these answers changed as a result of specific scenarios in the vignettes.

References

1 Ingvarsson S, Augustsson H, Hasson H, *et al.* Why do they do it? A grounded theory study of the use of low-value care among primary health care physicians. *Implementation Science*. 2020;15:93–93. doi: 10.1186/s13012-020-01052-5

2 Augustsson H, Ingvarsson S, Nilsen P, *et al.* Determinants for the use and de-implementation of low-value care in health care: a scoping review. *Implementation Science Communications*. 2021;2:13–13. doi: 10.1186/s43058-021-00110-3

3 Dulmen S van, Naaktgeboren CA, Heus P, *et al.* Barriers and facilitators to reduce low-value care: a qualitative evidence synthesis. *BMJ Open*. 2020;10:e040025. doi: 10.1136/bmjopen-2020-040025

4 Beatty PC, Willis GB. Research Synthesis: The Practice of Cognitive Interviewing. *Public Opinion Quarterly*. 2007;71:287–311. doi: 10.1093/poq/nfm006
