# Supplementary material for: Relative importance and interactions of factors influencing low-value care provision: a factorial survey experiment among Swedish primary care physicians
Source: BMJ Qual Saf. 2025 Feb 13;34(9):e018045. doi: 10.1136/bmjqs-2024-018045 (PMC12418588; doi:10.1136/bmjqs-2024-018045)
Supplement: online supplemental material 3 [file bmjqs-34-9-s003.pdf]

Supplementary material 3 – Factorial design and sampling of the vignettes

In this experiment, we aimed to investigate the effects of six main factors and selected interactions (i.e., those between patient request with remaining factors and those between credibility of evidence with other factors) on the outcome of interest. Out of the vignette universe consisting of 3 x 2 x 2 x 2 x 2 x 2 = 96 vignettes, we chose only a subset of the possible combinations. This constituted *fractional factorial design*. Reducing the number of combinations is typical for factorial survey experiments [1], making the study more feasible. It was also appropriate because in our research questions we focused on estimating main effects (each factor on its own) and only a sub-sample of lower-order interactions, as presented above.

In order to select a fraction of the vignettes for an optimal design, we decided to use D-efficient designs, which offer multiple advantages over the earlier approach of random selection from the vignette universe (see [2] for detailed discussion). The goal was to ensure a well-distributed representation of all factor levels (level balance) and chosen interactions, as well as reduce the risk of collinearity by means of ensuring orthogonality (all main effects and chosen interaction effects can be estimated uncorrelated) [1,2]. For this purpose, we used a statistical optimization algorithm to select a subset of vignettes that maximizes the precision of estimated effects. This was done applying the SAS macro '%MktBlock' to choose and distribute the vignettes across the decks, ensuring the effects of interest are possible to estimate. As suggested, we orthogonalized all two-way interactions (applying the so-called Resolution V design; [1]). This way, the algorithm ensured a well-distributed representation of all factor levels and chosen interactions, reducing the risk of collinearity. Based on the D-efficiency values (a standardized measure in the range from 0 to 100 of the goodness of fit of the designs; [1]), a design with a D-efficiency of 98/100 was deemed the best among all other combinations suggested by the algorithm. Below, we present the combinations of factors

(levels) that were used in each vignette and in each of the four decks as specified by the algorithm.

Table S3.1: Distribution of factor levels across the vignettes blocked in four decks

		Vignette factors					
**Deck**	**Vignette**	**Patient age**	**Patient request**	**Physician perception**	**Evidence credibility**	**Time consumption**	**Cost**
1	1	1	1	1	2	1	1
	2	1	1	2	2	1	2
	3	2	2	1	1	2	1
	4	2	2	2	2	1	1
	5	3	1	1	1	2	2
	6	3	2	2	1	2	2
2	1	1	2	1	1	1	2
	2	1	2	1	2	2	2
	3	2	1	2	1	1	1
	4	2	1	2	2	2	2
	5	3	1	2	2	2	1
	6	3	2	1	1	1	1
3	1	1	1	1	1	2	1
	2	1	2	2	2	2	1
	3	2	2	1	2	1	2
	4	2	2	2	1	2	2
	5	3	1	1	2	1	2
	6	3	1	2	1	1	1
4	1	1	1	2	1	2	2
	2	1	2	2	1	1	1
	3	2	1	1	1	1	2
	4	2	1	1	2	2	1
	5	3	2	1	2	2	1
	6	3	2	2	2	1	2

References

1 Auspurg K, Hinz T. *Factorial Survey Experiments*. Thousand Oaks: SAGE Publications, Inc. 2015.

2 Dülmer H. The Factorial Survey: Design Selection and its Impact on Reliability and Internal Validity. *Sociological Methods & Research*. 2016;45:304–47. doi: 10.1177/0049124115582269
