# Supplementary material for: Relative importance and interactions of factors influencing low-value care provision: a factorial survey experiment among Swedish primary care physicians
Source: BMJ Qual Saf. 2025 Feb 13;34(9):e018045. doi: 10.1136/bmjqs-2024-018045 (PMC12418588; doi:10.1136/bmjqs-2024-018045)
Supplement: online supplemental material 4 [file bmjqs-34-9-s004.pdf]

Supplementary material 4 – Simple effects analyses across all significant interactions in Model 2

Interactions — Post-hoc tests with Bonferroni correction

patient request x evidence credibility

comparison									
**patient request**	**evidence credibility**		**patient request**	**evidence credibility**	**difference**	**SE**	**t**	**df**	**$p_{bonferroni}$**
present	unconvincing	-	present	convincing	11.06	1.02	10.81	2943	< .001
present	unconvincing	-	absent	convincing	22.85	0.99	23.12	2820	< .001
absent	convincing	-	present	convincing	-11.79	1.03	-11.50	2861	< .001
absent	unconvincing	-	present	convincing	-8.26	1.68	-4.91	2945	< .001
absent	unconvincing	-	present	unconvincing	-19.32	1.65	-11.71	2944	< .001
absent	unconvincing	-	absent	convincing	3.53	1.59	2.22	2944	0.157

patient request x physician's perception of the practice

comparison									
**patient request**	**physician's perception of the practice**		**patient request**	**physician's perception of the practice**	**difference**	**SE**	**t**	**df**	**$p_{bonferroni}$**
present	negative	-	present	positive	-11.38	1.26	-9.05	2801	< .001
present	negative	-	absent	positive	1.35	1.17	1.15	2937	1
absent	negative	-	present	negative	-18.38	1.35	-13.61	2942	< .001
absent	negative	-	present	positive	-29.76	1.19	-25.09	2921	< .001
absent	negative	-	absent	positive	-17.03	1.44	-11.86	2893	< .001
absent	positive	-	present	positive	-12.73	1.75	-7.29	2748	< .001

patient request x time associated with the practice

comparison

patient request	time associated with the practice		patient request	time associated with the practice	difference	SE	t	df	$p_{bonferroni}$
present	not time-consuming	-	present	time-consuming	18.7	2.28	8.20	1991	< .001
present	not time-consuming	-	absent	time-consuming	21.18	1.80	11.74	2944	< .001
absent	not time-consuming	-	present	not time-consuming	-28.63	2.68	-10.69	1712	< .001
absent	not time-consuming	-	present	time-consuming	-9.93	1.15	-8.66	2785	< .001
absent	not time-consuming	-	absent	time-consuming	-7.45	2.82	-2.64	2010	0.05
absent	time-consuming	-	present	time-consuming	-2.48	2.33	-1.06	2224	1

patient request x cost of the practice

	comparison								
**patient request**	**cost of the practice**		**patient request**	**cost of the practice**	**difference**	**SE**	**t**	**df**	$p_{bonferroni}$
present	low	-	present	high	6.81	2.22	3.06	2027	0.013
present	low	-	absent	high	20.21	1.86	10.84	2138	< .001
absent	high	-	present	high	-13.40	1.37	-9.81	2936	< .001
absent	low	-	present	high	-10.91	2.52	-4.32	1904	< .001
absent	low	-	present	low	-17.71	1.15	-15.40	2924	< .001
absent	low	-	absent	high	2.49	2.02	1.23	1779	1

patient request x patient age

	comparison								
**patient request**	**age**		**patient request**	**age**	**difference**	**SE**	**t**	**df**	$p_{bonferroni}$
present	25	-	present	50	0.21	2.06	0.10	2625	1
present	25	-	present	75	3.37	2.39	1.41	1662	1
present	25	-	absent	50	16.33	1.29	12.70	2421	< .001
present	25	-	absent	75	9.14	1.50	6.09	2758	< .001
present	50	-	present	75	3.16	1.64	1.92	2574	0.819
present	50	-	absent	75	8.93	1.36	6.57	2921	< .001

absent	25	-	present	25	-24.78	4.09	-6.06	2386	< .001
absent	25	-	present	50	-24.57	2.76	-8.89	2650	< .001
absent	25	-	present	75	-21.41	2.76	-7.76	2939	< .001
absent	25	-	absent	50	-8.45	3.71	-2.28	2737	0.342
absent	25	-	absent	75	-15.64	3.19	-4.91	2513	< .001
absent	50	-	present	50	-16.12	1.91	-8.43	2944	< .001
absent	50	-	present	75	-12.96	1.83	-7.08	2295	< .001
absent	50	-	absent	75	-7.18	1.47	-4.89	2944	< .001
absent	75	-	present	75	-5.77	1.74	-3.32	1978	0.014

evidence credibility x cost of the practice

**comparison**									
**evidence credibility**	**cost of the practice**		**evidence credibility**	**cost of the practice**	**difference**	**SE**	**t**	**df**	**$p_{bonferroni}$**
convincing	low	-	convincing	high	5.35	1.91	2.80	2126	0.031
convincing	low	-	unconvincing	high	-2.65	2.29	-1.16	1943	1
unconvincing	high	-	convincing	high	7.99	1.68	4.75	2933	< .001
unconvincing	low	-	convincing	high	11.94	2.10	5.69	2066	< .001
unconvincing	low	-	convincing	low	6.60	1.11	5.93	2942	< .001
unconvincing	low	-	unconvincing	high	3.95	2.55	1.55	1962	0.724
