# Supplementary material for: Relative importance and interactions of factors influencing low-value care provision: a factorial survey experiment among Swedish primary care physicians
Source: BMJ Qual Saf. 2025 Feb 13;34(9):e018045. doi: 10.1136/bmjqs-2024-018045 (PMC12418588; doi:10.1136/bmjqs-2024-018045)
Supplement: online supplemental material 5 [file bmjqs-34-9-s005.pdf]

Supplementary material 5 - Distribution of respondents across Swedish regions

Table 5.1: Distribution of respondents across Swedish regions

Region	Number	% of Total
Blekinge	10	1.7%
Dalarna	15	2.6%
Gotland	6	1.0%
Gävleborg	14	2.4%
Halland	18	3.1%
Jämtland Härjedalen	11	1.9%
Jönköpings län	24	4.2%
Kalmar län	8	1.4%
Kronoberg	10	1.7%
Norrbotten	13	2.3%
Skåne	67	11.7%
Stockholm	131	22.8%
Sörmland	18	3.1%
Uppsala	32	5.6%
Värmland	23	4.0%
Västerbotten	7	1.2%
Västernorrland	7	1.2%
Västmanland	15	2.6%
Västra Götalands	101	17.6%
Örebro län	14	2.4%
Östergötland	31	5.4%
Missing data	*18*	*3.0%*

Note. Percentages are based on the total number of respondents (N = 593).
